# Toll-Like Receptor 4 and Blood Pressure: Lessons From Animal Studies

**DOI:** 10.3389/fphys.2019.00655

**Published:** 2019-05-29

**Authors:** Kenia Pedrosa Nunes, Amanda Almeida de Oliveira, Victor Vitorino Lima, R. Clinton Webb

**Affiliations:** ^1^Department of Biomedical and Chemical Engineering and Sciences, Florida Institute of Technology, Melbourne, FL, United States; ^2^Institute of Biological and Health Sciences, Federal University of Mato Grosso, Barra do Garças, Brazil; ^3^Department of Physiology, Medical College of Georgia at Augusta University, Augusta, GA, United States

**Keywords:** TLR4, blood pressure, animal models, hypertension, immune system

## Introduction

Hypertension affects nearly 1.2 billion people worldwide and challenges health systems with its myriad complications. While the molecular mechanisms underlying this disease are yet to be defined, the literature suggests abnormal immune system activation participating in its pathophysiology (Schiffrin, 2013; Norlander et al., 2018). Dysfunctional innate immune stimulation, primarily via Toll-like receptors (TLRs), plays an essential part in hypertension. Evidence suggests that these receptors act as gateways contributing to chronic low-grade inflammation, oxidative stress, and vascular remodeling (McCarthy et al., 2014). Out of thirteen characterized TLRs, TLR4 is the most explored in hypertension. Pharmacological blockade of TLR4 decreases oxidative stress and inflammation in the cardiovascular, renal, and central nervous systems, which blunts vascular hypertensive effects and reduces organ damage (Biancardi et al., 2017; Nunes et al., 2019). Despite these favorable outcomes, it is still not clear how TLR4 affects blood pressure (BP) under normal and hypertensive conditions.

BP regulation occurs through an intricate and complex network of interrelated systems (for review Touyz, 2014). Thus, to clarify the role of TLR4 in BP control under normal and hypertensive conditions, distinct animal models have been used in the last decade. This receptor has been targeted not only through pharmacological agents but also by genetic approaches. TLR4 blockade was performed locally, systemically, and centrally, aiming at understanding its roles in the labyrinth of pathways that are disrupted during hypertension. In previous work, we explored the effects of systemic TLR4 blockade with an anti-TLR4 antibody, and we demonstrated lower BP in spontaneously hypertensive rats (SHR) (Bomfim et al., 2012) and deoxycorticosterone acetate (DOCA)-salt rats (Nunes et al., 2012), but not in angiotensin (Ang) II-infused animals (Nunes et al., 2017). Further exploring the literature, we also observed discordant results regarding BP, when targeting TLR4, in different hypertensive models (Table 1). Therefore, we discuss evidence supporting the controversial roles of TLR4 in regulating BP in hypertensive animal models to further speculate possible physiological mechanisms that might be associated with the discrepant results shown in the current literature, which in turn, might enlighten future approaches in this field.

**Table 1:** Divergence of blood pressure regulation in response to pharmacological blockade or genetic deletion of Toll-like receptor 4 in animal models of hypertension.

**Hypertensive model**	**Animal**	**Type of blockade**	**Outcome**	**References**
Doca-salt (200 mg/kg for 6 weeks)	Male Sprague Dawley rats	anti-TLR4 antibody (1 μg/day for 21 days; i.p.)	Reduced MAP in DOCA-salt without affecting baseline levels in CTL animals	Nunes et al., 2012
AngII-infused (200 ng/kg/min for 14 days)	—	Knockout TLR4–/–	TLR4^−/−^ prevented MAP increase in animals injected with AngII	Dange et al., 2013
AngII-infused (90 ng/kg/min for 28 days)	Male C57BL/6 mice	anti-TLR4 antibody (1μg/day for 14 days; i.p.)	It did not affect MAP in AngII-infused neither in CTL animals	Nunes et al., 2017
AngII-infused (1.1 mg/kg/day for 14 days)	Male Balb/c and TLR4-deficient mice	TLR4*^*lps*/*d*^*	It did not affect SBP in AngII-infused neither in CTL animals	Nakashima et al., 2015
AngII-infused (1 μg/kg/min for 14 days)	—	TLR4–/– in cardiomyocytes	TLR4^−/−^ in cardiomyocytes prevented MAP increase in animals infused with AngII without affecting baseline levels in vehicle treated	Sriramula et al., 2016
AngII-infused (200 ng/kg/min for 14 days)	Male Sprague Dawley rats	VIPER (40 μg/kg/day for 14 days; i.c.v.)	Prevented increase in MAP in animals infused with AngII	Dange et al., 2014
AngII-infused (1.1 mg/kg/day for 14 days)	Male Balb/c and TLR4-deficient mice	TLR4*^*lps*/*d*^*	It did not affect SBP in AngII-infused neither in CTL animals	Matsuda et al., 2015
Aldo-salt (1 mg/kg/day for 28 days)	Male Wistar rats	TAK-242 (2 mg/kg/day for 28 days; s.c.)	Reduced SBP and DBP in Aldo-salt animals.	Zhang et al., 2015
SHR	Male Wistar rats and SHR	anti-TLR4 antibody (1 μg/day for 15 days; i.p.)	Reduced MAP in SHR animals without affecting baseline levels in CTL animals.	Bomfim et al., 2012
SHR	Male Wistar rats and SHR	VIPER (40 μg/kg/day for 14 days; bilateral canulae into the PVN)	Reduced MAP in SHR animals	Dange et al., 2015
L-NAME (50 mg/kg/day for 14 days)	C56BL/6 mice	TLR4–/–	Prevented increase in SBP and DBP in animals	Sollinger et al., 2013

*TLR4, Toll-like receptor 4; MAP, mean arterial pressure; WT, wild type; CTL, control; CNS, central nervous system; SBP, systolic blood pressure; DBP, diastolic blood pressure; SHR, spontaneously hypertensive rats; L-NAME, L-N^G^-Nitroarginine methyl ester; VIPER, viral inhibitory peptide of TLR4; –, data not possible to be accurately extracted from the paper; i.p., intraperitoneal; s.c., subcutaneous; i.c.v., intracerebroventricular; PVN, paraventricular nucleus*.

## Differential Modulation of BP in Animal Models of Hypertension by TLR4 Blockade

**Figure 1:**
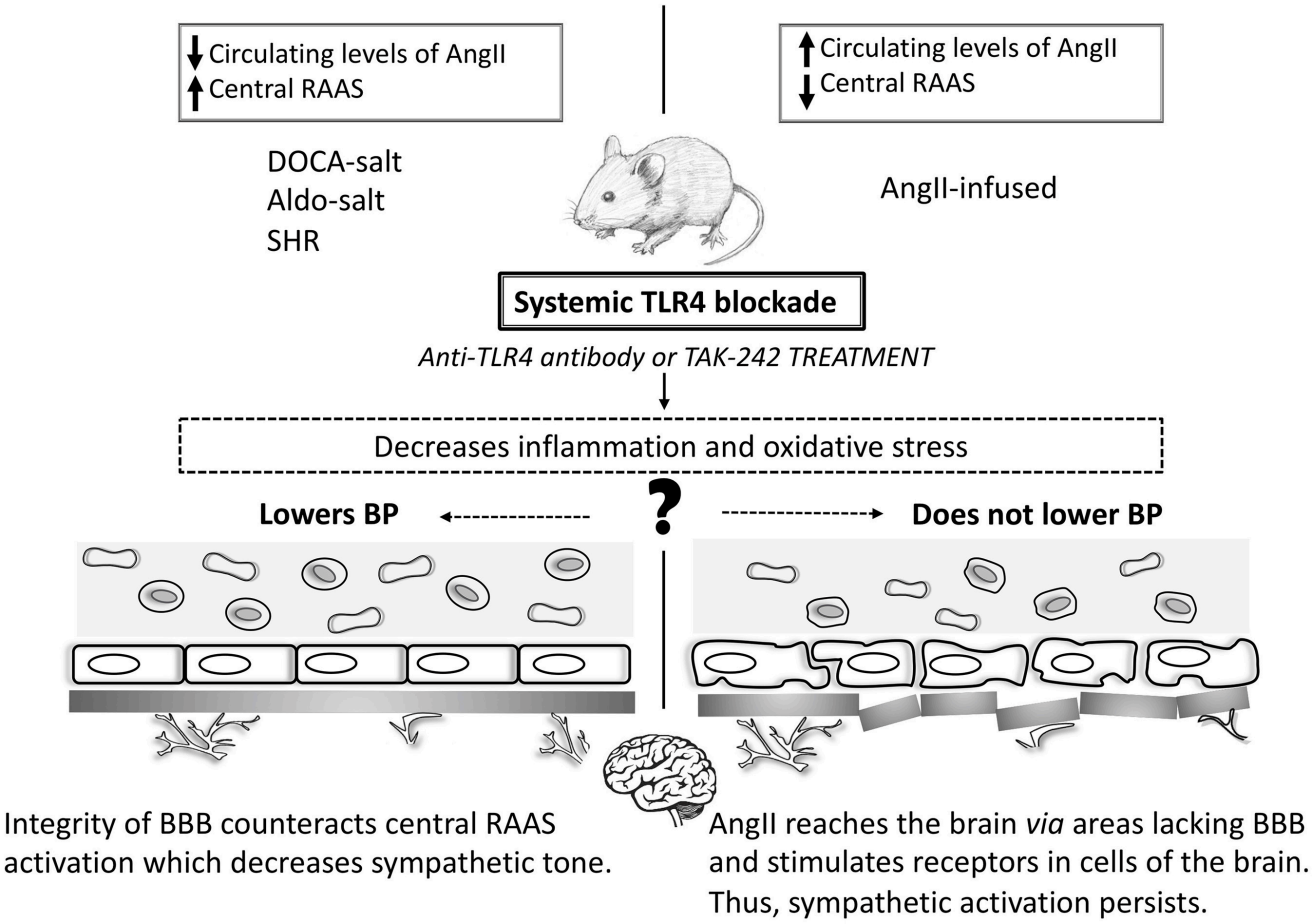
Toll-like receptor 4 and blood pressure in animal models of hypertension. In animal models where systemic TLR4 blockade lowers BP (DOCA-salt, Aldo-salt, and SHR) the improvement of the BBB counteracts the central RAAS system, which decreases the sympathetic tone. However, in AngII-infused animals, AngII reaches the brain through areas lacking the BBB and stimulates receptors in cells of the brain. Thus, sympathetic activation persists, and BP does not decrease. Central TLR4 blockade lowers BP in all studied animal models of hypertension. TLR4: Toll-like receptor 4; BP: blood pressure; SHR: spontaneously hypertensive rats; RAAS: renin-angiotensin-aldosterone system; AngII: angiotensin II.

Mounting evidence suggests TLR4-mediated low-grade inflammation as a critical unifying mechanism in the major BP controlling mechanisms (McCarthy et al., 2014; Bomfim et al., 2017; Nunes et al., 2019). Still, the modulation of TLR4 in hypertensive animal models produces different effects on BP (Table 1; Figure 1). Notice that comparison of hypertensive murine models is not entirely equitable as BP responses in rat and mouse are somewhat different and may involve unknown mechanisms. Nevertheless, it is crucial to discuss possible underlying mechanisms linking TLR4 to BP regulation in the current literature, especially with the promising, but challenging, opportunity to target TLR4 or its downstream signaling adaptors (MyD88 or TRIF) to manage vascular pathologies such as hypertension.

### DOCA-Salt, Aldo-Salt, and SHR Animals

We have previously reported lowering of BP in DOCA-salt animals treated with an anti-TLR4 antibody (Nunes et al., 2012). Considered a neuroendocrine model, these animals have disrupted renin-to-aldosterone ratio, increased sympathetic nerve activity, augmented levels of AngII in areas of the brain related to BP regulation, and disrupted immune response (Nishimura et al., 1999; Beuschlein, 2010; Iyer et al., 2010; Grobe et al., 2011; Wenzel et al., 2016; Basting and Lazartigues, 2017). Additionally, a massive inflammatory process is also observed in these animals, which contributes to vascular damage (Ko et al., 2006). Although the DOCA-salt model mimics an aggressive stage of hypertension, it still shares many pathophysiological characteristics with other hypertensive models and represents an essential tool for uncovering molecular mechanisms of organ damage during chronic sustained hypertension (Basting and Lazartigues, 2017). Moreover, augmented mineralocorticoid activity is increasingly documented in patients with resistant hypertension and so-called low-renin hypertension, considering the efficiency of mineralocorticoid receptor (MR) blockade in these patients (Ferrario and Schiffrin, 2015). In another mineralocorticoid-dependent hypertensive model, aldosterone and salt administration (Aldo-salt), it was demonstrated that TLR4 expression increases in cardiac and renal tissues (Zhang et al., 2015). In these animals, treatment with a TLR4 signaling antagonist (TAK-242) inhibited inflammatory cytokines and decreased BP. In SHR, a genetic model of hypertension with both neural and vascular alterations (Harwani et al., 2012; Leong et al., 2015), our group previously reported similar effects regarding lowering BP after long-term treatment with an anti-TLR4 antibody (Bomfim et al., 2012). In this model, the brain RAS plays a crucial role in the development and establishment of the hypertensive state (Phillips et al., 1977). In fact, reduced central AngII content and decreased activation of its downstream pathways were suggested to be an important mechanisms to maintain BBB integrity in SHR (Buttler et al., 2017). In these animals, inhibition of TLR4 within the paraventricular nucleus (PVN) with a viral inhibitory peptide also decreases BP, suggesting mechanistic evidence that TLR4 might regulate the hypertensive response within the central nervous system (CNS) (Dange et al., 2015). Indeed, TLR4 blockade buffers neuroinflammation (Lehnardt et al., 2003; Guo et al., 2017) further affecting the regulation of cardiovascular parameters (Dinh et al., 2014; Winklewski et al., 2015).

DOCA-salt, Aldo-salt, and SHR are models of neurogenic hypertension reported to have increased levels of AngII in the CNS and disruption of the blood-brain barrier (BBB). The BBB physically separates the CNS from the peripheral circulation avoiding the paracellular movement of substrates in this environment (Setiadi et al., 2018). Recent findings have supported that a crosstalk between AngII and TLR4 happens via the AngII type I receptor (AT1r) in microglial cells (Biancardi et al., 2015). Indeed, in these animals AT1r blockade prevents BP increase by reducing TLR4 levels in microglial cells, which might provide a role for this pathway in the mechanisms of sympathoexcitation-associated neurogenic hypertension (Mowry et al., 2018). Additionally, these animal models represent forms of hypertension that may have a portion of BP elevation due to endogenous ouabain-like substances (Leenen, 2014). Over the last decade, it has become clear that the turning point for increasing the activity of angiotensinergic signaling pathways within the brain is partially dependent on the endogenous ouabain pathway. Ouabain can be synthesized by the hypothalamus (Murrell et al., 2005) and binds to Na, K-ATPase membrane protein in hippocampal astrocytes, activating inositol triphosphate receptor (InsP3R), which leads to calcium oscillations, thus activating NF-κB (Liu et al., 2007). Likewise, ouabain was suggested to stimulate formation of protein complex including TLR4, which indirectly regulates inflammatory cytokines (Chen et al., 2017). Furthermore, throughout the brain renin-angiotensin-aldosterone system (RAAS), increased central levels of aldosterone might activate MR, which elevate intracellular calcium signaling leading to enhanced ouabain-like substances and, consequently, sympathoexcitation (Leenen, 2010). Noteworthy, increased sympathetic outflow is not only a likely outcome of high BP but also a triggering mechanism (Fisher and Paton, 2012). Another potential connection between these animals is the disruption of the BBB, which directly associates with the levels of inflammatory cytokines and oxidative stress. Disturbance in the BBB has been associated with autonomic nervous system dysregulation (Buttler et al., 2017) and, eventually, cardiovascular dysfunction (Biancardi et al., 2014; Setiadi et al., 2017), which could help explain why systemically TLR4 blockade attenuates BP in these animals. Additionally, BBB disruption is associated with leukocyte infiltration that further differentiates into microglial cells to produce inflammatory cytokines, which ultimately leads to neuroinflammation (Setiadi et al., 2018). Likewise, altered BBB can also be targeted in the luminal side by circulating inflammatory mediators produced during sustained hypertension as well as by inflammatory factors derived from the neurovascular unit such as the glial component. Following analyses of the outcomes produced by TLR4 blockade in these animals, we speculate that: (a) enhanced sympathetic outflow may be associated with BBB disruption; (b) reduced sympathetic activity lowers BP; and, (c) that attenuation of inflammation and oxidative stress following systemic TLR4 blockade might contribute to preserving the integrity of the BBB, which was suggested to be crucial for maintaining the central autonomic control.

### Angiotensin II-Infused Animals

In AngII-infused animals, outcomes regarding BP were not as apparent as the ones observed in the models mentioned above. Systemic TLR4 blockade does not protect against BP elevation in AngII-dependent hypertensive animals (Nunes et al., 2017), except when TLR4 is antagonized within the PVN (Dange et al., 2014), suggesting that central TLR4 plays an important role in BP regulation. In this case, even though systemic TLR4 blockade decreases pro-inflammatory mediators and improves vascular function, which contributes to keeping the integrity of the BBB, the high circulating levels of AngII might be stimulating the central RAAS (Yao and May, 2013; Biancardi et al., 2014). While circulating AngII is too large for crossing the BBB, components of the angiotensinergic-sympathoexcitatory pathways are also located in areas of the brain lacking this structure (Biancardi and Stern, 2016). These unique neural regions act as ways through which systemic AngII can mediate the autonomic response during pathological conditions. Thus, one could say that in AngII-infused animals, circulating levels of this peptide might be amplifying the sympathetic tone *via* crosstalk with the local brain RAAS. This crosstalk is partially dependent on the functionality of the central TLR4 (Biancardi et al., 2015). Furthermore, an association between AngII and TLR4 in microglial cells, the brain's innate immune cells, has been previously demonstrated (Biancardi et al., 2015). These cells are considered to be the core of the innate immune system in the brain. Additionally, microglial cells are ubiquitously expressed in the sympathetic nervous system and are capable of modulating sympathoexcitation (Kapoor et al., 2016). More importantly, BBB disruption allows peripherally circulating factors such as AngII to enter the CNS (hypothalamus, brainstem, and PVN) (Biancardi et al., 2014; Buttler et al., 2017), to influence downstream signaling pathways. Interestingly, AngII is believed to mediate BBB leakage through binding the AT1r on endothelial cells, which are the chief component of the barrier. Activation of AT1r within the CNS triggers oxidative stress and inflammation, dual critical components that are present in sustained high BP. Corroborating this theory, while systemic TLR4 blockade in AngII-infused animals does not lower BP, its central inhibition is an effective mechanism in BP control (Dange et al., 2014). The interplay between TLR4 and AngII contributing to BBB disruption are critically intertwined, and mainly undefined. However, considering the current literature, two mechanisms might be considered here: TLR4 activation via AngII/AT1r crosstalk (Yang et al., 2009) and/or AngII as a direct agonist for TLR4 via MD2 (Han et al., 2017).

### Knockout Animals

Cardiomyocyte-specific TLR4 deletion decreases BP in AngII-infused mice (Sriramula et al., 2016), suggesting a role for cardiac TLR4 in BP regulation. It has been previously demonstrated that congenic lack of TLR4 (B6.B10ScN-*Tlr4*^*lps*−*del*^/JthJ) contributes to cardiovascular function by upregulation of the parasympathetic branch of the autonomic nervous system. Indeed, TLR4 knockout mice have an increased parasympathetic tone, which decreases cardiac output and, consequently, BP (Okun et al., 2014). Indeed, TLR4 deletion protects mice infused with AngII against BP elevation, which suggests that AngII effects in BP are partly mediated through this receptor (Dange et al., 2013). Similarly, mice lacking the gene for TLR4 are protected against increase in BP in response to L-N^G^-Nitroarginine methyl ester (Sollinger et al., 2013). Interesting, previous work showed that downstream TLR4, deletion of one of its co-adaptors, MyD88 or TRIF, leads to opposing effects in BP. More specifically, only TRIF knockout animals were protected against BP elevation stimulated by AngII infusion (Singh et al., 2015). Further analyzing the literature, we also observed that animals with a deficiency in TLR4^*lps*−*d*^ infused with AngII had no effects in BP (Matsuda et al., 2015; Nakashima et al., 2015). Notice that because the TLR4 deficiency in these animals is restricted to lipopolysaccharide activation, it is unknown whether this receptor is still responding to enhanced AngII levels in the same manner.

## Final considerations

In summary, while the idea of TLR4 involvement in BP regulation is gathering broader support, we are only now uncovering pathways mediated by this receptor during hypertension. As discussed here, TLR4-mediated BP regulation appears to be tissue specific and to oscillate accordingly to the diseased model. At this point, the literature supports the notion that central or cardiac blockade of TLR4 attenuates BP without affecting baseline levels. It seems that the effects of TLR4 in BP regulation are associated with activation of angiotensinergic-sympathoexcitatory pathways within the CNS and disruption of the BBB might be a critical event in this process, especially in animals where hypertension is induced independently of the circulating levels of AngII. Nevertheless, further studies are needed in order to confirm the roles of TLR4 in BP regulation as well as whether this process is linked to the integrity of the BBB, which could leverage our understanding of autonomic diseases, especially hypertension.

## Author Contributions

All authors listed have made a substantial, direct and intellectual contribution to the work, and approved it for publication.

### Conflict of Interest Statement

The authors declare that the research was conducted in the absence of any commercial or financial relationships that could be construed as a potential conflict of interest.
